# Charge-Trapping-Induced Hysteresis Effects in Highly Doped Silicon Metal–Oxide–Semiconductor Structures

**DOI:** 10.3390/ma15082733

**Published:** 2022-04-08

**Authors:** Piotr Wiśniewski, Bogdan Majkusiak

**Affiliations:** 1 Centre for Advanced Materials and Technologies CEZAMAT, Warsaw University of Technology, 02-822 Warsaw, Poland; 2 Center for Terahertz Research and Applications (CENTERA), Institute of High-Pressure Physics, Polish Academy of Sciences, 01-142 Warsaw, Poland; 3 Institute of Microelectronics and Optoelectronics, Warsaw University of Technology, 00-662 Warsaw, Poland; b.majkusiak@imio.pw.edu.pl

**Keywords:** traps, hysteresis, metal-insulator-semiconductor, tunneling spectroscopy, semiconductor devices

## Abstract

It is shown that a simple metal–oxide–semiconductor (MOS) structure with highly doped silicon substrate can exhibit current–voltage hysteresis effects related to sudden rises and drops in the flowing electric current. Experimental current–voltage characteristics of Al-SiO_2_-(n++Si) structures are presented and discussed. Their analysis shows that the ohmic and shallow traps assisted space-charge limited conduction (SCLC) are the dominating transport mechanisms. Sudden rises and drops in the flowing current, leading to the current–voltage hysteresis effects, are attributed to tunneling through deep traps in the oxide. Based on inelastic electron tunneling spectroscopy (IETS), the energy levels of the deep traps and their position in the oxide are evaluated.

## 1. Introduction

Semiconductor devices exhibiting nonlinearities or hysteresis on the current–voltage dependence are of great interest for their potential applications in electronics, e.g., signal generation or detection, and memory functions. The hysteretic behavior of a device is usually associated with physical effects such as ferroelectricity, magnetoresistance, resistive switching, and spin-phenomena [1,2,3,4], etc. Some works show that current–voltage (I-V) hysteresis can also occur due to resonant tunneling in metal–oxide–semiconductor (MOS) structures [5,6,7]. The behavior of devices exhibiting current–voltage characteristics with steps, peaks, or negative differential resistance is usually attributed to tunneling or resonant tunneling [8]. In our previous work [9], we observed such behavior in highly doped n-type silicon MOS structures and proposed to explain sudden jumps in the current at the negative gate bias direction by the resonant tunneling of electrons from the semiconductor conduction band through the semiconductor bandgap, surface inversion region, and the insulator bandgap, to the metal conduction band. However, such a mechanism cannot explain sudden drops in the flowing current at the positive gate bias direction, leading to current–voltage hysteresis. Moreover, the investigated structures were undertaken in a proper electroforming process, and exhibited resistive switching, as presented in another work [10]. This work analyzes the current–voltage characteristics of Al/SiO_2_/n++ Si structures not subjected to the forming process, and explains their behavior using electron tunneling spectroscopy [11]. This is a very sensitive technique that can be used to study the thin gate dielectrics and can provide lots of valuable information, particularly about the location of traps [12]. We show that the shape of the I-V curves can be attributed to the space-charge limited conduction (SCLC) related to shallow traps and tunneling through deep trap levels in the oxide.

## 2. Materials and Methods

**Figure 1:**
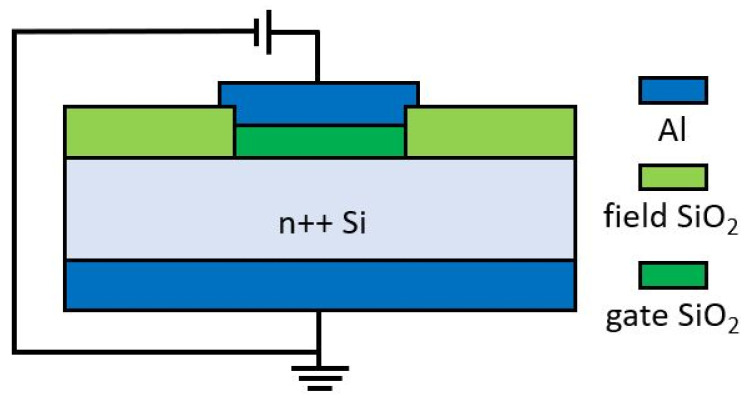
Schematic picture of the measured structure (not to scale).

The MIS structures were fabricated using the standard CMOS-compatible processes. 2″ n-type highly doped (arsenic, resistivity less than 0.005 Ωcm) wafers were cleaned using the standard RCA method, and then, the wet oxidation process was carried out in a high-temperature furnace Thermco 2803 Furnace System. A photolithography process and wet etching were used to open windows in the field oxide. Wet etching was performed in a BHF solution. Then, 5–6 nm thin gate oxide was grown in a dry oxidation process (10 min at 820 °C). In the end, the Al top gate and bottom contact layers were deposited in magnetron sputtering processes and formed in the appropriate photolithography and etching processes. Structures were annealed in H_2_/Ar atmosphere at 400 °C for 30 min. A schematic picture of the fabricated structure is presented in Figure 1. Measurements were carried out using the Keithley 4200-SCS Semiconductor Characterization System (Keithley Instruments, LLC, Solon, OH, USA) with a Süss MicroTec PM8 low noise probe shield. DC measurements were performed with the static source-measure unit (SMU) connected to the device under test at room temperature. All studied devices were fabricated on the same wafer.

## 3. Results and Discussion

**Figure 2:**
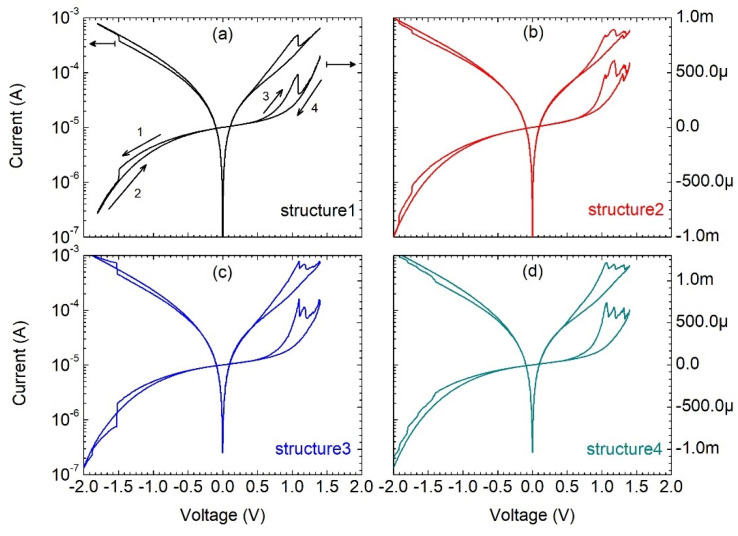
Current–voltage characteristics of Al/SiO_2_/n++ Si structures (**a**–**d**) with a gate diameter of 156 um.

As discussed in the literature, transport through a dielectric can be controlled by various mechanisms [13,14,15], e.g., Poole–Frenkel, TAT, direct tunneling, Fowler–Nordheim tunneling, etc. Figure 2 shows the measured current–voltage characteristics in the logarithmic and linear scales for four different structures of the 156 μm gate diameter. The numbered arrows in Figure 2a indicate the measurement sequence.

The devices present similar electrical characteristics exhibiting current–voltage hysteresis with a different number of features—sudden current rises at the negative gate voltages and current drops at the positive gate voltages. When the gate voltage increases, the current jumps to a new current–voltage dependence. The number of observed current rises at the negative gate bias coincides with the number of current drops at the positive gate bias.

**Figure 3:**
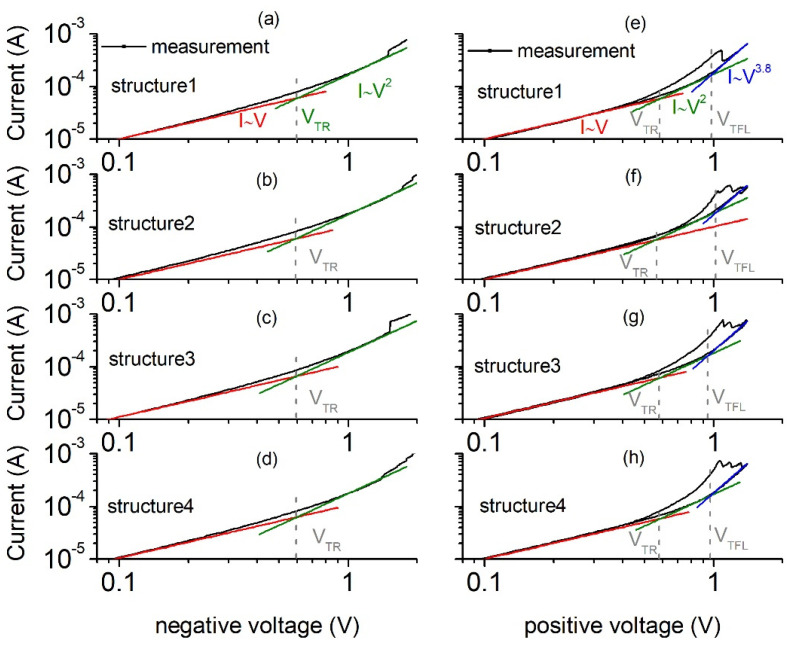
Slope of the current–voltage characteristics in the log-log scale for different Al/SiO_2_/n++ Si structures for the negative (**a**–**d**) and positive (**e**–**h**) voltages.

In order to examine the transport mechanism in the studied structures, the I-V characteristics of the same structures are plotted in Figure 3 in the log-log scale, together with fits of the slope in different voltage ranges. At the low voltage range, the current is proportional to the voltage, identifying the ohmic transport mechanism, whereas it changes to quadratic dependence at a given transition voltage *V_TR_* for both the positive and negative gate voltage polarizations. The parabolic dependence is related to the space-charge limited conduction through a region containing partially filled shallow traps [16]. Traps are being filled up, and only a fraction of the injected carriers enter the conduction band and contribute to the current. When increasing the positive voltage above *V_TFL_*, one can observe a higher-order current–voltage dependence (I~V^3.8^) for all measured structures. Fermi level moves upwards to near the conduction band of the dielectric, which results in an increase in the slope of the I-V dependence [15,17]. According to the SCLC theory with single-level shallow-trap centers, one can express the ohmic conduction current (*J_Ohmic_*) at low voltages and the trap-filling component (*J_TFL_*) by the following formulas

(1) $$J_{Ohmic}=qn_{0}\mu \frac{V}{d}$$

(2) $$J_{TFL}=\frac{9}{8}\mu \varepsilon \theta \frac{V^{2}}{d^{3}}$$

where *q* is the electron charge constant, *n*_0_ is the free charge carrier concentration at thermal equilibrium, *μ* is the electron mobility in the dielectric, *V* is the applied voltage, *d* is the dielectric thickness, *ε* is the electric permittivity, and *θ* is the ratio of the free carrier density to the total carrier density (free and trapped). The *V_TR_* and *V_TFL_* voltages, which are marked in Figure 3, are given by

(3) $$V_{TR}=\frac{8}{9}\frac{qn_{0}d^{2}}{\varepsilon \theta }$$

(4) $$V_{TFL}=\frac{qN_{t}d^{2}}{2\varepsilon }$$

where *N_t_* is the trap concentration in the dielectric. Values of *V_TR_* and *V_TFL_* for the considered structures and the concentration of traps in the oxide, extracted from Equation (4), are presented in Table 1.

It is worth noticing that values of the trap concentration which were obtained for the positive voltages for different structures are similar. We do not observe *V_TFL_* transition points at negative voltages. Instead, sudden single or multiple current rises are observed, which cannot be explained using SCLC theory. We also observe current drops at the positive voltages. We suspect that such behavior might be related to the transport through deep trap levels instead of shallow traps.

**Figure 4:**
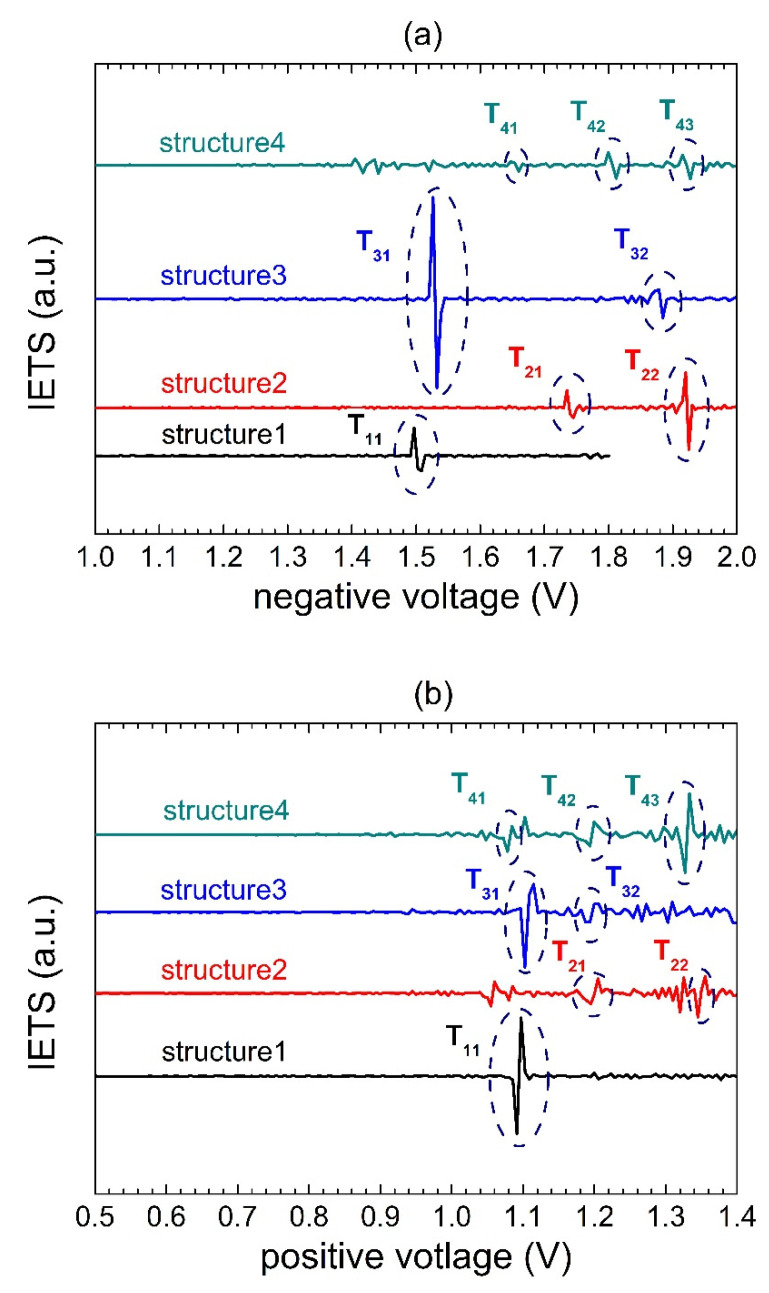
IETS signal of Al/SiO_2_/n++ Si structures for the negative (**a**) and positive (**b**) voltages.

In order to understand these features, we analyzed the transport through the traps using inelastic electron tunneling spectroscopy (IETS). It is a technique that can be used to study phonons, bonding vibrations, and impurities [12,18] in MIM/MIS structures. It can also be helpful to analyze tunneling through traps in dielectrics [19,20], giving information about their geometrical position and energy. The IETS signal is proportional to the current’s second derivative, and it is usually measured using the lock-in amplifier to reduce the signal-to-noise ratio. However, the observed features are clearly strong in our case, and we calculated the IETS signal straightforwardly from the I-V curves. The results are presented in Figure 4.

According to IETS theory [21], the shape of the signal can be related to the trap-assisted tunneling process or to the charge-trapping process. The relevant features are marked in Figure 4. We observe peak-to-valley shape signals associated with trap-assisted tunneling at the negative voltage values. Their occurrence corresponds to the voltages at which sudden current bumps appear in Figure 3a–d. For the positive voltages, we observe the valley-to-peak shape signals, which are usually attributed to the charge-trapping process [12]. We attribute these features rather to the blockage of the trap-assisted tunneling path, as will be shown later. Other less distinct features are visible in Figure 4 at lower voltages, which we ascribe to the shallow traps related to SCLC transport.

It has been shown [22] that positions of the peak-to-valley and valley-to-peak signals are related to the position *x_t_* of traps within the dielectric (the effective electrical distance from the oxide/metal gate interface) and the relative energy of the deep trap level *E_t_* = *qV_t_* above the Fermi level at the zero bias

(5) $$x_{t}=\frac{t_{ox}V_{p}}{V_{p}+V_{n}}$$

(6) $$V_{t}=\frac{V_{p}V_{n}}{V_{p}+V_{n}}$$

where *V_p_* and *V_n_* are the absolute values of the voltages for the positive and negative gate bias, respectively, at which the corresponding trap features are observed, and *t_ox_* is the oxide thickness. Table 2 shows the values of *x_t_* and *E_t_*, obtained for the investigated structures using Equations (5) and (6).

A number of trap levels can differ for different structures. Irrespective of this, the relative trap position does not change significantly, regardless of the trap energy, as well as the energy levels of the deep traps being similar for different structures. In order to analyze the I-V hysteresis and explain sudden current changes at the *V_p_* and *V_n_* points, the energy band diagrams of the investigated structure were calculated by using a 1D Poisson–Schrödinger solver. We assumed *t_ox_* = 5.5 nm, whereas other parameters used in electrostatics simulations were typical for the Al/SiO_2_/Si system.

**Figure 5:**
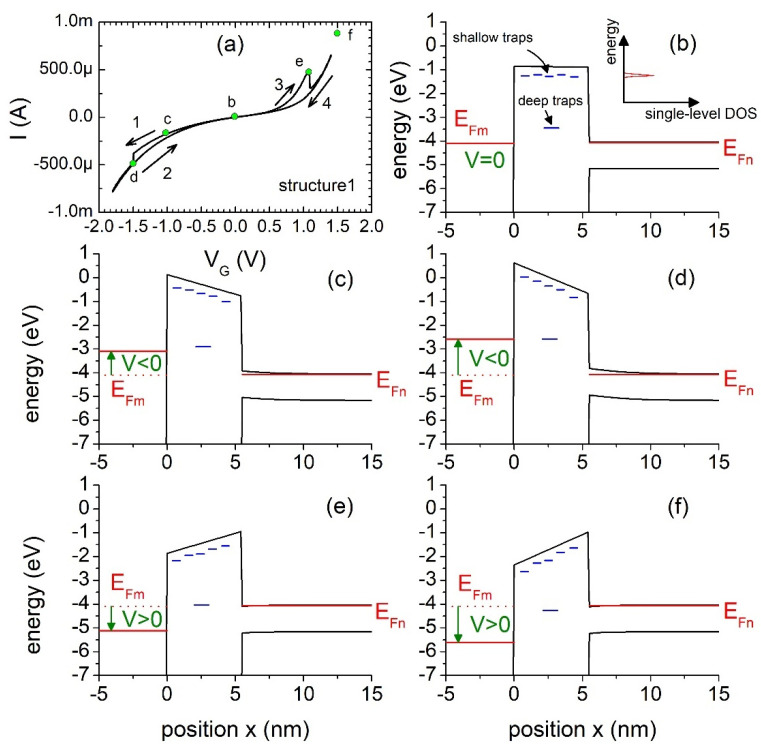
Energy band diagrams of the MIS structure no. 1 with marked trap energy levels in the dielectric for *V* = 0 (**b**), *V* < 0 (**c**,**d**) and *V* > 0 (**e**,**f**). Shallow trap levels are shown schematically, whereas the deep trap energy level position is calculated. Points corresponding to the band diagrams were marked in (**a**).

Figure 5 shows the energy band diagrams calculated for different bias conditions. Shallow trap levels participating in the SCLC transport are schematically added. We also added a deep trap energy level T_11_ calculated for structure no. 1. At sufficiently high negative voltage bias, one can observe that the position of the metal Fermi level E_Fm_ coincides with the position of the energy trap level. As a result, a sudden increase in the current is observed due to the possibility of the trap-assisted tunneling of electrons from the metal gate to the semiconductor conduction band. The electron charge is accumulated in the deep traps, and the charge de-trapping process is long enough to sustain the electrostatic effect during measurements. Thus, a different current–voltage dependence is observed when going back from the negative bias range to the positive bias range. The charge stored in the traps can contribute to the total current via tunneling to the metal or the semiconductor regions, provided that free states are available. Moreover, trap-assisted tunneling can also occur at a positive bias. At a given voltage *V_p_*, a sudden current drop related to the blockage of the trap-assisted tunneling path can be observed. Such a situation is depicted in Figure 5e. The deep trap energy level position aligns with the bottom of the conduction band. At a slightly higher gate voltage, the energy level *E_t_* lies in the range of the semiconductor bandgap, and trap-assisted tunneling is forbidden. The SCLC current dominates the deep trap-assisted tunneling current at small bias voltages (|*V*| < 0.7 V), regardless of the bias direction. The trapped charges modify the potential barrier electrostatically and affect the resultant transport process.

In our opinion, the proposed mechanisms can explain the hysteresis effects observed on the current–voltage characteristics of the investigated devices very well. The traps in the SiO_2_ layer might come from the silicon oxidation process of a highly doped silicon substrate. We have not observed the hysteresis effect in structures fabricated with the same process flow on wafers with low doping levels (ρ = 1–10 Ωcm). A very high doping level has a pronounced effect on the oxidation rate and the resultant material stress during the process. Moreover, due to the redistribution of dopants during the oxidation process, the formed oxide can absorb the dopants from silicon, causing material defects. All these factors can affect the material quality and be the origin of traps.

## 4. Conclusions

This work shows that SiO_2_-based metal–insulator–semiconductor devices with highly doped silicon substrates can exhibit hysteresis effects. We analyzed the current–voltage characteristics of the fabricated devices and identified the transport mechanisms. We evaluated the trap concentration and identified deep trap energy levels and their position within the dielectric layer. We showed that I-V hysteresis could be related to the trap-assisted tunneling through these defects and the charge trapping effect.

## Figures and Tables

**Table 1:** Parameters obtained from I-V measurements, based on SCLC theory for different structures.

Structure	*V_TR−_* (V)	*V_TR+_* (V)	*V_TFL_* (V)	*N_t_* (cm^−3^)
1	0.606	0.594	0.969	1.38 × 10^19^
2	0.590	0.555	1.033	1.47 × 10^19^
3	0.598	0.583	0.945	1.35 × 10^19^
4	0.601	0.586	0.969	1.38 × 10^19^

**Table 2:** Relative position and energy of deep trap levels for different structures.

Structure	#Trap Level	*V_p_* (V)	*V_n_* (V)	*x_t_*/*t_ox_*	*E_t_* (eV)
1	T_11_	1.11	1.51	0.42	0.64
2	T_21_	1.20	1.74	0.41	0.71
	T_22_	1.34	1.92	0.41	0.79
3	T_31_	1.11	1.53	0.42	0.64
	T_32_	1.20	1.88	0.39	0.73
4	T_41_	1.08	1.66	0.39	0.65
	T_42_	1.20	1.82	0.40	0.72
	T_43_	1.33	1.92	0.41	0.79

## Data Availability

The data presented in this study are available on request from the corresponding author.
